# Post-Graduation Teaching in London

**Published:** 1903-11-28

**Authors:** 


### POST-GRADUATION TEACHING IN LONDON.

It is a matter of fairly widespread notoriety that as a clinical centre for post-graduation study London has largely failed to exercise any considerable attractive force. The failure is all the more conspicuous in view of the enormous material and the distinguished professional names of which the metropolis may legitimately boast. Attractive in itself for historical, patriotic and social reasons, London has not proved itself able to develop a scheme of post-graduation study and opportunity anything like equal to its civic pre-eminence. So far as facilities of communication with the outside world are concerned its opportunities are enormous. Equally the speech of its schools opens it without difficulty to the profession in all English-speaking lands. Yet it remains true that whilst medical men from the colonies and the United States may be found in numbers as students at more than one continental capital, they can only be counted as occasional units in the greatest city in the world. Even British practitioners are hardly tempted by the opportunities of their own metropolis. To them also Vienna, Paris, and Berlin can make a stronger and more profitable appeal. Further, it is not in the least improbable, in view of recent developments, that a strongly attractive influence will in the immediate future be exercised by the large schemes now coming to maturity on the other side of the Atlantic. To those familiar with the progress of events in the United States the recent prophecy of a distinguished physician that the medical graduate of the future will seek his more advanced training in the land of the setting sun will seem the reverse of a rash expectation. Regarding these positive achievements in other lands there can only be sentiments of satisfaction and congratulation. But inevitably the question must arise why London lags in the rear. The complete answer would involve an analysis of many professional and social conditions, some of them in present circumstances possibly beyond substantial modification. But all will agree that one element responsible for the defective development of London's enormous possibilities as a field for clinical study is the want of organisation and co-ordination. There are, no doubt, difficulties caused by the wide area over which the hospitals are scattered. Possibly there is some conflict of interests, real or imagined. But these and others would doubtless disappear in the presence of a scheme by which a really worthy school for post-graduation work would be evolved. What is to be desired are opportunities for clinical observation, for instruction in special methods of diagnosis and treatment, and for practical laboratory tuition. These, no doubt, exist already, but they exist as isolated attempts and not as parts of a definite and coherent plan. As a result they severally fail, because no one of them covers the whole field or has the commanding position necessary to arrest attention. Each scheme has its own limited programme, which, so far as it goes, may be very good, but the spirit is too often parochial and contracted, and in any case lacks the inspiration and dignity of a large and organised effort. Even in medical schools and among distinguished physicians and surgeons men may readily mistake the hum of their burgh for the murmur of the world. It is necessary to remember that London as a medical centre is something much more than the sum of the so-called teaching hospitals. The University of London already carries the burden of many hopes, and to these it may be permitted to add the anticipation that in some not unduly distant future, when the age of attainment has been reached, it may include among its schemes the direction and development of a scheme of post-graduation study and research. It is not research alone that is required, valuable as everyone admits this to be. Provision is needed for the general practitioner and the young graduate who desire to train themselves in modern methods and to enlarge and revive their clinical experiences. In these respects we are glad to recognise the work that is being done by such institutions as the West London Hospital and the Polyclinic and Medical Graduates' College. These organisations have not only the merit of present performance, but the promise of considerable development in the future. Guided by worthy motives and sound policy they may prove themselves the starting points of a movement which will raise London as a post-graduation school to the proud position it ought long since to have attained.

